# What can be done to reduce the prevalence of teen pregnancy in rural Eastern Uganda?: multi-stakeholder perceptions

**DOI:** 10.1186/s12978-020-00984-x

**Published:** 2020-08-31

**Authors:** Josephine Nabugoomu, Gloria K. Seruwagi, Rhona Hanning

**Affiliations:** 1 School of Public Health and Systems, University of Waterloo, 200 University Avenue West, Waterloo, ON N2L 3G1 Canada; 2 Makerere University School of Public Health, P. O. Box 7062, Kampala, Uganda

**Keywords:** Pregnancy in adolescence, Social cognitive theory, Developing countries, Health planning, Capacity building

## Abstract

**Introduction:**

The teenage pregnancy rate of 25% in Uganda is worrying though it may seem low compared to 28% in Sub-Saharan countries and West and Central Africa. Young mothers in Uganda risk poor maternal and child health, being isolated, attempting unsafe abortions, failure to continue with school, and poverty. This paper describes perceptions and recommendations of young mothers, family and community members on why the high rate of teenage pregnancies in Uganda and how these can be reduced.

**Methods:**

This qualitative research was conducted from March to May 2016 in six communities within Budondo sub-county (Jinja district), Eastern Uganda. In-depth oral interviews were conducted with 101 purposively sampled adolescent mothers, family members, and workers of government and non-government organizations. Thematic analysis framed around levels of influence within a social cognitive framework was conducted using Atlas-ti (version 7.5.4).

**Results:**

Perceived determinants of teenage pregnancies include: lack of life and social survival skills, lack of knowledge on how to avoid pregnancy, low acceptance/use of contraceptives, neglect by parents, sexual abuse, pressure to contribute to family welfare through early marriage or sexual transactions, lack of community responsibility, media influence, peer pressure, cultural beliefs that promote early marriage/childbearing and lack of role models. Other contributing factors include drug use among boys, poverty, late work hours, long travel distances, e.g., to school, and unsupervised locations like sugarcane plantation thickets. Recommendations participants offered include: sensitization seminars and counselling for parents and girls, closing pornography outlets that accept entrance of minors, using the law to punish rapists, involvement of the President to campaign against early pregnancies, school dismissal before dark, locally accessible schools and job creation for parents to earn money to support the girls financially. Areas for capacity building are: training teachers and community members in transferring empowerment and vocational skills to girls, and construction of homes with separate rooms to support parents’ privacy.

**Conclusion:**

The factors associated with adolescent pregnancy in Uganda fall under individual, economic, social and physical environmental determinants. Recommendations spanning family, community and government involvement can ultimately empower girls, their families and community members, and support collective action to reduce teenage pregnancies.

## Plain English summary

**Introduction** The high prevalence of adolescent motherhood in Uganda and the region of Busoga in particular is worrying and raises public health concerns. This paper describes perceptions and recommendations of community stakeholders about what can be done to reduce the prevalence of teen pregnancies.

**Methods** This qualitative research was conducted in six communities within Budondo sub-county (Jinja district), Eastern Uganda. In-depth interviews were conducted with 101 adolescent mothers, family members and community workers. A thematic analysis using Atlas-ti (version 7.5.4) influenced by the social cognitive theory was conducted.

**Results** Perceived determinants of teenage pregnancies include: lack of social skills, lack of knowledge on how to avoid pregnancy, low acceptance/use of contraceptives, neglect by parents, sexual abuse, lack of community responsibility, media influence, peer pressure, cultural beliefs that promote early marriage and lack of role models. Other factors include drug use among boys, poverty, late work hours, and long travel distances, e.g., to school. Recommendations suggested include: counselling for parents and girls, closing pornography outlets that admit students, punishing rapists, President’s campaign against early pregnancies, school dismissal before dark, accessible schools and job creation for parents to support the girls financially. Areas for capacity building are: training leaders of students and school alumni to counsel youth, and training teachers in girls’ empowerment and vocational skills.

**Conclusion** The factors associated with adolescent pregnancy in Uganda fall under individual, economic, social and physical environmental determinants. Recommendations spanning family, community and government involvement can ultimately empower girls and reduce teenage pregnancies.

## Background

Teenage pregnancies are higher in developing countries than developed countries, and more so with in the sub-Saharan Africa 28% of adolescents give birth before the age of 18 [1]. Twenty eight percent (28%) of girls in West and Central Africa have had a live birth by the age of 18 while Eastern and Southern Africa has 25% [1]. In Uganda, more than one out of four adolescents (15–19 years) become pregnant with the rates being higher (27%) in rural than urban Uganda (19%) [2]. This is a particular issue in the Busoga region of eastern Uganda [2], raising public health concerns [1, 3, 4]. Although little has been documented about Eastern Uganda in particular, factors contributing to increased teenage pregnancies in Uganda are: high fertility rate, risky sexual behaviors, peer pressure into early sex, child marriages, lack of education, lack of family support, low socio-economic status [2–8], low education levels [2, 9] and low use of contraceptives [2]. The risks to young mothers of poor maternal/child health [10–12], may be exacerbated by stigmatization, isolation from family, unsafe abortion attempts, and lack of adolescent maternal-friendly services at health centers [9]. Adolescent mothers are also at a high risk of poverty since those who don’t drop out of school may be sent away from school [9], and others lose their employment – all contributing to reduced potential for gainful employment [13]. Some of the interventions that are seen as avenues to reduce teenage pregnancies in Africa include: availability and accessibility of quality education (both academic and sexual), keeping or re-enrollment of young mothers in school, and counselling and guiding students to make the right choice of keeping in school [1]. According to WHO, interventions must be combined and integrated for effectiveness [1]. Uganda is committed to ending child, early and forced marriages by year 2030 through: co-sponsoring the 2013 and 2014 UN General Assembly and 2013 Human Rights Council resolutions and on early and forced marriages [14]. Uganda also set the age of marriage at 18 and in 2015, the same country launched the African Union Campaign to end child marriage [14]. Policies, strategies, campaigns and sensitizations by the Ministry of Gender and Labor and Social Development in conjunction with organizations under the “GIRLS NOT BRIDES” partnership and UN agencies all aim at ending early marriages and child bearing [14].

In the course of a broader study exploring needs, barriers and opportunities for improved health and nutrition of teen mothers, community stakeholders including parents, family members, teachers, health-related personnel, local political leaders, religious leaders and other service providers) invariably wanted to discuss factors associated with the high prevalence of teen pregnancy and how this might be reduced. The purpose of this study was, therefore, to describe determinants of adolescent pregnancies in Jinja District from the perspectives of a range of community stakeholders and their recommendations to address the issue.

## Methodology

**Fig. 1:**
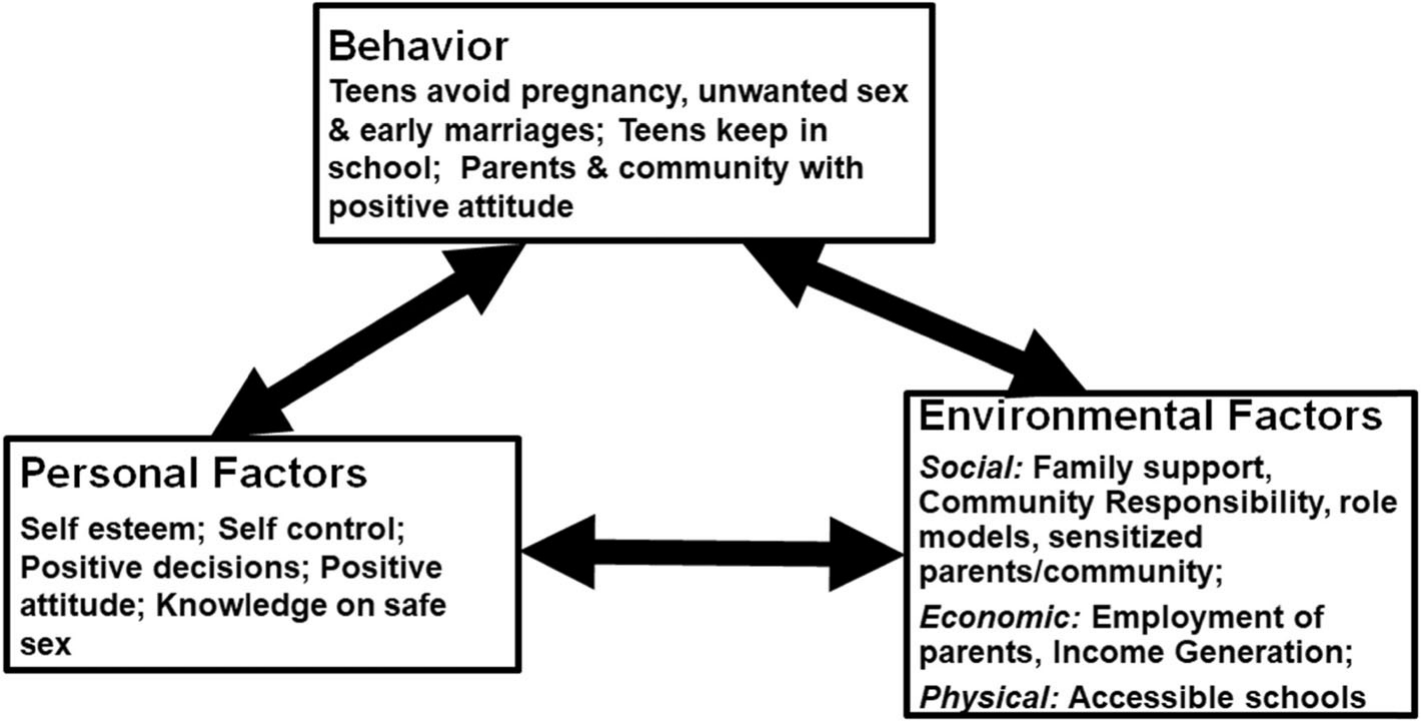
Social Cognitive Theory Framework of Determinants of Teenage Pregnancies, Recommendations for Action and Areas of Capacity Building to Reduce Teenage Pregnancies. *Adapted by Authors from:* Glanz K, Rimer BK, Viswanath K. Health behavior and health education: Theory, research, and practice. 2008. Jossey-Bass. Fourth Edition. p 42, 169, 170, 273, 274. http://riskybusiness.web.unc.edu/files/2015/01/Health-Behavior-and-Health-Education.pdf

The social cognitive theory (SCT) (Fig. 1) was adopted as the framework for this research, to help understand how individual factors and environmental factors, including social, economic and political factors, interact reciprocally with behaviors [15–18] of teenagers to contribute to early pregnancies. The study bent towards the epistemological stance of post-positivism so as to emphasize community participant perspectives and reduce any bias from the researcher’s perceptions or background knowledge [19–25]. For in-depth deliberations, the study interviews used open ended questions [20, 26] combined with close ended questions or probes built on levels of influence within the SCT [19–25].

### Study site

The study was carried out from March to May 2016 in rural Busoga region of Eastern Uganda, Jinja district, Budondo sub-county [27]. The sub-county has an estimated 36.3% [28] of its population of 51,560 [27] living below the poverty line [28].

### Inclusion criteria

Study participants were eligible for recruitment if they had resided within Budondo sub-county or Jinja district for at least 3 years and signed study consent forms. Young mothers (10–19 years) had to have their first pregnancy or a first baby under 12 month, and have been in school in the study area within the previous 3 years.

### Study sample and recruitment

With an aim of representing adolescent mothers, family members and community service providers within each of the 6 communities of Budondo sub-county, 101 study participants (see Table 1) were mobilized by 6 study coordinators who were community VHTs (Village Health Team workers) through purposive sampling [20, 29, 30] by assessing eligibility and inviting those participants who were either family members or community service providers who closely related/worked with young mothers. Study invitation and consent letters translated into Lusoga language were read to the illiterate participants by the 6 VHTs. For all the young mothers who participated in this study, the VHTs made sure there were adult parents or family members who gave verbal and written for parental consent at invitation to participate in the study and before interviews. The study VHTs who witnessed the consent processes also co-signed the participants’ consent forms. Illiterate adult study participants were able to give their verbal and written consent. The signed consent letters are safely locked away in the Corresponding Author’s office at the University of Waterloo, Waterloo, Ontario, Canada. The administrators at health centers, schools, and sub-county and district headquarters whose interviews were conducted in English were not given invitation letters as invitation was done by mouth for short interviews which took less than 10 min.

**Table 1:** Demographics of study respondents (*N* = 101)

Respondent Category	Gender		Number
	Male	Female	
Young Mothers	0	25	25
Family Members	0	11	11
Teachers	0	5	5
Head teachers	9	2	11
Health-related Personnel	5	14	10
Agricultural Officers	3	0	3
Religious Leaders	3	0	3
Local Council I (LCI) Chairpersons	6	0	6
District Administrators	4	4	8
Sub-county Administrators	3	2	5
NGO Staff	3	2	5
**Total**	**35**	**66**	**101**

### Data collection

Data collection took place from March to May 2016. Interview guides were translated into Lusoga language and delivered by the researcher (JN) aided by local trained research assistants. Before use in the study areas, oral questions were tested in rural Butagaya sub-county with those typical of the target groups. At the start of each oral interview, participants were welcomed by the researcher, and fully informed about the study aims and ethical consideration like confidentiality of their contributions. Each interview was conducted in privacy at the homes or work places of participants, taking an average of 40 min.

### Data analysis

**Fig. 2:**
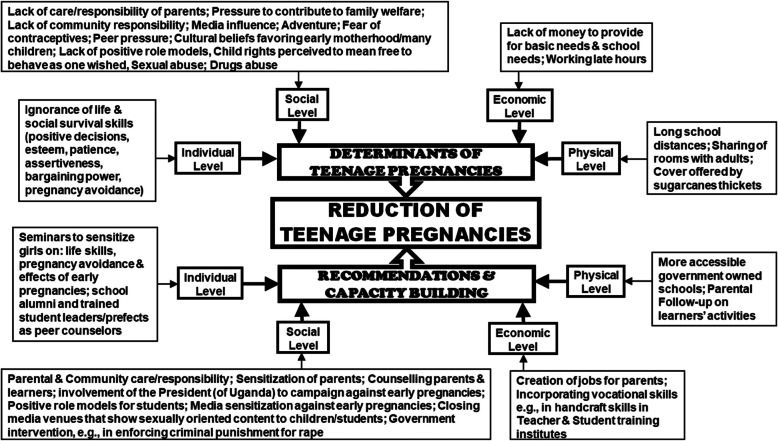
Thematic Network of Determinants of Teenage Pregnancies, Recommendations for Action and Areas of Capacity Building to Reduce Teenage Pregnancies. *Adapted by Authors from:* Attride-Stirling J. Thematic networks: an analytic tool for qualitative research. Commission for health improvement, England. Qualitative Research. *SAGE Publications* (London, Thousand Oaks, CA and New Delhi). 2001;1(3):385–405

Interview recordings were transcribed and translated into English then coded on the basis of SCT levels and emerging codes related to determinants of early pregnancies and recommendations for action or capacity building to reduce teenage pregnancies. Using Atlas.ti 7.5.4 software, phrases were coded and underwent thematic analysis [20, 30–33] as shown in Fig. 2.

## Results

### Demographics of the respondents

Table 1 shows the demographic characteristics of study participants. Twenty five percent of the study respondents were adolescent mothers aged 13–19. The number of other study participants include: 11 family members of the young mothers; 19 health related personnel; 16 school teachers and principals; 19 leaders at the local council, sub-county and district; 3 agriculture workers, 3 religious leaders; and 5 staff members of area NGOs. The young mothers who participated in this study were unmarried. Family members of married/cohabiting teenage mothers who had agreed to participate in the study refused them to go on with the study while the fathers of their infant were not willing to give in their views. Although young mothers who were married/cohabiting and fathers of their infants refused to participate in the study, the views given by other young mothers, family and community members could be illustrative of what is happening in the communities.

### Perceived determinants of adolescent pregnancies, and recommendations for action and capacity building to reduce teenage pregnancies

Stakeholders revealed determinants of pregnancy and suggested recommendations and areas for capacity building that were categorized by individual, social, economic and physical levels.

### Individual level

#### Determinants of teenage pregnancies at the individual level

It was perceived that girls in the rural areas of Uganda lacked life and social skills such as making positive decisions, self-esteem, patience, assertiveness and a bargaining power which could help them better navigate life’s challenges and avoid early sex and or early pregnancies. For example, respondents pointed out that teenagers admired some items provided by boys/men. In turn, these boys/men demanded sex and it was perceived that the girls felt that they could not say no. Some girls were perceived to welcome the attention of boys, as in the quote below, for intimate/sexual relationships.

*“Sometimes you may need some money but when your parents cannot raise it and a boy comes providing for everything. When he wants you to pay back, what can you use to pay back his money if not sex since you are not working?”* Young Mother 1.

*“Girls no longer fear boys, they start chasing for the boys from like when they are 10 years old so what do you expect boys to do when the girls show them that they want to have sex. Girls do not say no at all, when the boys propose something, they just go with them.”* Health-related Personnel 1.

It was also perceived that girls were ignorant about the ways to avoid pregnancy including the use of contraceptives.

*“We* [adolescent girls] *do not know when it is most risky to get pregnant and we just rely on chance not to get pregnant which lets us* [girls] *down. Is it safer to have sex before or after menstruation? How do we count those days* [of the menstrual cycle] *exactly? We also do not know how to use contraceptives yet we fear asking the Nurses who treat us harshly because use of contraceptives at our young age is not acceptable in our communities.”* Young Mother 2.

#### Recommendations to reduce teenage pregnancies at individual level

Stakeholders recommended use of seminars to sensitize children with the aim of improving their life and social survival skills.

*“Children should be openly talked to by all of us about the effects of early pregnancies and pre-marital sex and not just giving in to the pleasure of sex that lasts for a short time yet the effects are long lasting. They have to learn to say no to early sex”* Family Member 1.

#### Capacity building to reduce teenage pregnancies at individual level

Stakeholders recommended use of school alumni and trained school student leaders (also known as prefects) as motivators and peer counselors, respectively, to empower girls and boys into avoiding early sex and prioritizing education.

*“Schools should call back old students and also train all leaders of students into becoming good role models so as to counsel and empower both girls and boys to firmly say no to early sex but instead study their books.”* Sub-county Administrator 1.

### Social environment level

#### Determinants of teenage pregnancies at the social level

It was perceived that adolescent girls in the rural areas of Uganda were mostly impregnated by fellow school mates or youths who had dropped out of school and were working, for example, as motorbike (boda boda) riders, sugarcane harvesters, food and retail workers. Stakeholders suggested a number of social determinants of early pregnancy; these included: failure by teachers and medical personnel to teach learners about how to avoid pregnancy, such as abstinence and use of the safe days of their menstrual cycle, and perceived failure of parents to adequately educate or supervise their children.

*“I think the menstrual cycle education has been ignored* [in our schools] *because most teachers seem not to know what to say while those that may know are so shy about such a topic and besides they may be victims of early pregnancy themselves and so do not have the moral authority to talk about such issues. Even medical personnel can’t come to schools to help* [educate students]*, without facilitation* [money paid as allowances and for transport costs]*.”* Sub-county Administrator 2.

*“Most parents leave the responsibility of advising and guiding their children to the school. Some parents say ‘go to school, that is where you will be guided from’. Parents have that tendency and say that teachers are the ones who will manage their children. You can send them back home from school at 6 pm but you find them in the trading center at 9:00 pm in their school uniforms. However it should be that once this child gets home late, a responsible parent must question them where they have been but when the girls go back and they are not tasked, they consider it as something very normal and have left the children to whom-it-may-concern.”* Head teacher 1.

A number of other social determinants were also identified. Respondents described pressure to contribute to family welfare whereby girls were thought to have been pushed, or may have felt pushed, into sexually involving themselves with men who could give them money, for example, to buy some items like sugar for the family. There was also perceived lack of community responsibility, for example to question the behavior of a given teen. Moreover, lack of positive role models was another social determinant as some parents and peers were victims of early pregnancies hence, were perceived not to be able to stand firmly against teenage pregnancies. In addition, sexual abuse from fathers, male relatives and teachers; child rights that were felt to be misunderstood by children to mean ‘freedom’ to act as one wanted. Respondents also identified that acceptance of settling out of court by parents of the girls who become pregnant on being given money and failure to legally prosecute the male offenders encouraged boys/men to impregnate girls and yet continue in freedom. Girls fear to use contraceptives due to perceived negative side effects; and fear of reprisal from their parents and other community members who feel contraceptives are not acceptable based on their religious beliefs.

*“A grandmother can send a girl in the evening and tell her that we do not have sugar or food, please go and look around for it. Remember the girl has no shop or business so she looks for men* [to provide]*.”* Religious Leader 1.

*“But how would one avoid early pregnancy if your mother and elder sisters did the same? We are talking about the environment and the people they relate with. They do not have the right role models. Sadly, some girls are raped by their fathers, male relatives and teachers. Also how can pregnancies stop when the law isn’t tough on offenders, they just walk scot free knowing they will not be prosecuted besides some parents settle issues out of court after being given some little money”* NGO Staff 1.

*“As parents, we do not accept contraceptives, we are religious and so accepting these contraceptives means giving our children permission to have sex. What will we tell God on the judgment day? Moreover some contraceptive methods are said to have side effects like heavy bleeding and irregular periods which may be dangerous to them* [girls]*, yet they are still young. So even when the girls go to health centers, I am not sure if the Nurses are willing to help with that”* Religious Leader 2.

Media, such as pornography and other sexual content in movies, videos, song lyrics or on social media sites, was perceived to influence young people into trying out the sexual scenes or considering sex as an adventure or something their peers are doing. In addition, rape from young men, some under the influence of drugs, was described as a contributing factor. Cultural beliefs favoring early motherhood and a large number of children, commonly referred to as ‘children of the President of Uganda’ (His Excellency Yoweri Kaguta Museveni), were identified as contributing to the situation.

*“The boys and girls watch blue movies and immoral films so they practice what they see. The owners of these video halls are networked with highly placed governmental officials and if you touch them* [video owners]*, they* [government officials] *will call you to stop all operations. Also disco dancing, music shows and social media like Facebook and WhatsApp have made girls go off track”* Local Council I Chairperson 1.

*“Culture also affects them, here in Busoga people feel it is great to give birth while young and to give birth to many children and now they refer to them as* [President] *Museveni’s children and voters!”* Sub-county Administrator 3.

### Recommendations to reduce teenage pregnancies at the social level

Stakeholders recommended parental and community involvement to openly advise their children and not shy away, sensitization of parents to support (especially) the girl child, counselling of both parents and learners about effects of early pregnancy and the responsibilities that come with it, community care and responsibility to keep girls in school, and using the law to legally prosecute offenders.

*“Parents should openly talk to their children and tell them to abstain and inform them of the effects of early pregnancies and pre-marital sex without being shy. How can people just look on proudly saying those are President Museveni’s children. Museveni is not the father of those children being born and the earlier our girls and communities wake up to this reality the better. Museveni has many national issues to take care of and so everyone who gives birth should know it is their responsibility not for Museveni.”* District Administrator 1.

*“I remember when we were still at school, a child was looked after by the whole community which is not the case today. They worked together to make sure that students keep in school. If this was done as before, the girl child will be kept in school, thereby reducing their chances of getting pregnant while schooling. The boys/men who impregnate our girls should be strongly dealt with legally.”* Health-related Personnel 2.

Other recommendations included: use of the media in form of plays and talk shows to sensitize against early pregnancies, closing local media venues that show pornography and other sexually oriented content to children and students, using the law to punish rapists, and asking the office of the President (that has been so influential in supporting large families) to warn against early pregnancies.

*“Government should broadcast talks and plays about the disadvantages of early pregnancies over the televisions and radios.”* Teacher 1.

*“Government should close down all disco and video halls that show bad movies with sexual content especially those that admit school children.”* Family Member 2.

*“The culprits who rape or defile these girls should be taken to the courts of law and imprisoned. This is because others are HIV positive and want to infect our children. The President must add his strong voice to this by being tough to parents and children and refuse early pregnancies.”* Teacher 2.

### Capacity building to reduce teenage pregnancies at the social level

Stakeholders recommended training of teachers through colleges and in-service education sessions on how to empower girls, and about birth control methods.

*“Train all teachers at Primary Teacher Colleges and those in-service on how to counsel and empower the child to abstain and firmly say no to early sex.”* District Administrator 2.

*“Train teachers at institutes and in-service on birth control methods and empower them to boldly come out to tell children the real things and not be shy about them.”* District Administrator 3.

### Economic level

#### Determinants of teenage pregnancies at economic level

At the economic level, some parents in the rural areas of Uganda lack money to provide for basic needs and schools. It was suggested by respondents that parents may have encouraged girls to have men provide these needs or they sent their daughters to work late in the day, placing them at risk along the way back home. Economic pressures were thought to push some girls into prostitution and thereby risk of early pregnancies.

*“After they* [males] *give you money, they start demanding for sex. There are some needs I may have but when parents have no money or they give you little money and so that makes us to get pregnant. I may want clothes, shoes, panties, snacks, school fees but my parents can’t provide.”* Adolescent Mother 3.

*“Some parents send their children in the evening to go and sell some things like maize, ‘mandazi’ (fried leavened dough), pancakes in the trading centers. They come back late and boys take advantage of them with a high risk of early pregnancies.”* Local Council I Chairperson 2.

*“Some girls are promiscuous while others use sex to get money to survive especially orphans and those whose parents neglect them. They end up becoming pregnant”* Health-related Personnel 3.

#### Recommendations to reduce teenage pregnancies at economic level

It was suggested that government could create credit schemes and jobs for parents to help them financially support their children, and vocational training in schools to keep girls interested in schooling and help them acquire practical skills from which they could earn money.

*“Government should create jobs for those parents that are jobless so that they take care of their children adequately. They can also form groups which the government can finance to help lend parents money to create businesses.”* Agricultural Officer 1.

*“All schools in villages and towns should be facilitated by government to teach handcraft skills as these will break the monotony of academics thereby keeping our daughters interested in schooling. Moreover the girls can make these at home and earn a living instead of throwing themselves at men to support them financially. This issue of girls getting pregnant or looking for money from men is not only in villages, in fact it is more common in towns as some of the girls in town schools practice prostitution but they safely abort while many are on contraceptives. It is so degrading for our girls. If government cannot finance these skills as they say they do not have money, let the international organizations come up and finance such projects because this will help with future self-employment for the girls too.”* Head teacher 2.

#### Capacity building to reduce teenage pregnancies at economic level

It was suggested that teacher training institutions should incorporate different vocational skills in their curriculum so that qualifying teachers can train students who can then earn a living.

*“All student teachers while in their Institutes should be trained in many types of vocational skills like brick laying, plaiting hair, making briquettes, making jewelry and artwork so that they teach girls who can use the skills to earn a living.”* District Administrator 4.

### Physical environmental level

#### Determinants of teenage pregnancies at the physical level

Long distances to and from schools were thought to tempt girls to accept free rides from boda boda men (commercial bike drivers) and potentially place them at risk. Other factors identified included sharing of rooms with adults and thereby exposing them to overhearing sexual activity and environmental opportunity presented by sugarcane thickets.

*“A girl may be moving a long distance to school like St. Stephen Secondary School Budondo which is the only government and cheap secondary school and the boda boda men give them rides on their motor cycles. They also drop them home in the evening when they are coming back from school. How do you think these girls are going to pay for the rides since they do not have money?”* Agricultural Officer 2.

*“Some parents sleep in the same room with their children and so when they are having sex, their children are hearing so it causes the children to start those things* [having sex] *when they are still young hence becoming pregnant.”* Religious Leader 3.

*“We got a problem with those sugarcane plantations and when you walk there during noon time you will see many of them running out of there with girls …*. T*hey hide in there to have sex and so we refer to the sugarcane plantations as the lodges where those youths have sex.”* Health-related Personnel 4.

#### Recommendations to reduce teenage pregnancies at the physical level

Participants recommended construction of more local government owned schools and allowing earlier afternoon dismissal to facilitate student’s safe return home as physical environmental factors to help prevent early pregnancies.

*“Government should construct and facilitate public schools at primary and secondary level in each village so that girls do not move long distances. Students should study till 3:00pm so that they can go back home early to their parents.”* NGO Staff 2.

#### Capacity building to reduce teenage pregnancies at the physical level

It was suggested that parents and community leaders be trained in house construction using local yet strong materials to communally construct houses with different rooms for parents and children. Group credit schemes were suggested avenues to finance construction materials.

*“When we were growing up, our parents and community members used to get training and construct houses for each other in rounds. This needs to be re-introduced in our communities so that the houses for parents are big enough. Things like iron sheets can be bought with borrowings from group credit schemes which should be created. One can no longer stand on their own and manage.”* NGO Staff 3.

## Discussion

The study revealed a range of factors perceived to relate to early pregnancy and recommendations to reduce these determinants and associated teen pregnancies. Factors spanned individual, economic, social and physical levels and responses involved not only the girl child but also families, communities, and the government, underlining that teen pregnancy is a collective responsibility. Our study agrees with earlier studies on determinants of teenage pregnancies like: child marriages, lack of family support, peer pressure, low socio-economic status [1–8], and low use of contraceptives [2]. This study is strengthened by the large number of diverse stakeholders who participated. Nevertheless, we acknowledge the limitation that fathers of the infants and married or cohabiting teenage mothers did not participate, in spite of concerted efforts to include them.

This study was based on the social cognitive theory. Understanding the determinants of teenage pregnancy and what the community members closest to the issue suggest to reduce early pregnancies across a range of levels, may help to consider possible interventions. Participants in this study revealed that, as individuals, adolescents in the rural areas lacked life and social ‘survival’ skills such as making positive decisions, self-esteem, patience, assertiveness and a bargaining power which could help them survive through life’s challenges and avoid early sex and or pregnancies. Adolescent girls also lacked knowledge of how they could avoid pregnancy. Teenagers should be supported in making healthy choices in line with avoiding pregnancies by accessing health facilities which offer services and information related to sexual reproductive health e.g., counselling and contraceptive use [34]. School counsellors too could equip adolescents with coping skills to face challenges of life [35, 36], with cooperation and encouragement from parents and management of schools [37].

Advocating for support for young mothers from family and community members by the *Teenage Mothers Project* (TMP) in eastern Uganda, was successful in enrolling them back into schools [6, 38]. Information sharing and advocacy could also support a shift in parental/family member responsibilities in advocating for prevention of pregnancy; sexual and reproductive health education; collective stances against early marriages for girls; restricting exposure to pornography; and prohibiting use of sex for personal or family economic viability. Pregnancy is experienced biologically by females, but males in Uganda are often not held accountable and free to continue with education [14, 39, 40]. Many respondents, while recognizing collective community responsibility for the issue of teen pregnancy, inadvertently discussed the problem and solutions in relation to girls mainly.

The high prevalence of adolescent pregnancy occurs within a context in which there is a law against induced abortions [41–44] and low acceptance and use of contraceptives among female adolescents, possibly due to religious beliefs advocating for abstinence from sex among unmarried persons, as enforced by their families and communities, or due to lack of education about birth control to avoid unwanted pregnancies [45]. Culturally, while there are no data indicating religious beliefs specific to the study area, a large proportion of those living in rural parts of Uganda are Catholic (40.4%) and Anglican (32.8%) [27]. This suggests that many participants in the current study would follow these religions and opinions of the religious leaders [46]. While some Christian religious leaders have not clearly come out to support use of contraceptives among unmarried girls, they do embrace its use in marriage couples [47, 48], others have not embraced the use of contraceptives [48]. Religious leaders in Uganda also protested the inclusion of sexuality education in the primary and secondary schools saying it was against the country’s cultural norms and beliefs [49–52]. Another relevant aspect of context is that, in Uganda, it is a criminal offence to impregnate a girl under the age of 18 [9] but respondent identified that nothing happens to offenders who impregnate teenagers and communities may not be aware of where to run for help [39]. There is a clear need for better communication of the laws and processes for investigating and dealing with offenders. Through better communication and enforcement of laws, it is hoped that rates of teen pregnancies will diminish and support for affected teens improve.

Until such time when there is a shift in social, economic, physical and political environments, synchronizing of gender, education and support of both young girls and boys is especially important in avoiding early pregnancies. Prevention of teenage pregnancies would be enhanced by teachers and parents training in-school girls and boys in sexual and reproductive health. In addition, parents, family members, teachers, health-related personnel, local political leaders, religious leaders, and other community service providers should be skilled in sexual and reproductive health to enhance the fight against teen pregnancies. While it was not identified by participants in this study, government could revise the abortion law for safe abortions [41, 53] and women/girls to receive safe abortion services [41]. In addition, it is recommended that government and other stakeholders support adolescent friendly interventions for example, available and accessible information, counselling and services for comprehensive sexual and reproduction [34]. Community-based health facilities should provide a friendly, private and safe adolescent-centered environment for sexually active teenagers to comfortably receive timely service and free contraceptives to prevent unwanted pregnancies [34]. Accurate information and education on the different contraceptives have to be given to sexually active adolescents so that they make an informed choice [34]. Opinion leaders should be encouraged to accept that some teenagers are sexually active and embracing and advocating for the use of contraceptives [34].can help avoid abortions or teen pregnancies.

The Government of Uganda has put up a number of policies that should, in theory, be useful to reduce adolescent pregnancies including: setting the minimum age of sexual consent at 18 years; the defilement law against having sex with a girl under 18 years; Universal and Secondary Primary Education that offers free elementary and secondary education to children; and the National Adolescent Health Policy [9]. However, these policies do not seem to have much effect due to lack of enforcement and lack of knowledge about the policies by the general public. Similarly, the Uganda National Adolescent Health Policy, whose main goal is to emphasize adolescent health, wellbeing and equity, seems ineffective in the face of a culture that sends pregnant girls out of school [9].

External support through NGOs may also help parents and adolescents. Income generation support to parents is important because the present low socio-economic status of parents [54, 55] may not allow them to support their children’s financial needs. Microcredits for financial empowerment of parents could be lobbied for from the available microfinance deposit-taking institutions in Budondo sub-county or Jinja district, such as BRAC Uganda [56], Pride Microfinance Ltd. [57], Finca Uganda Ltd. [58], and Opportunity Bank [59]. While the aim is to support adolescent girls and allow them to continue in school even if pregnant, participants in this study felt that a range of options, including vocational training from trained teachers, might be helpful to support young girls and empower them to make positive choices. Income generation skills training and provision of resources, like seeds or animals to rear, are strategies that have worked in the area through NGOs, such as, SOUL Foundation^1^ [60] and PEFO^2^ [61].

## Conclusion

The factors associated with adolescent pregnancy in rural Uganda and suggested recommendations to reduce the high prevalence have been described in this paper, based on the input from a broad range of community stakeholders. While some shifts in social norms, physical environments, political support and economic wellbeing will take time, a number of feasible steps were identified. Reducing the prevalence and negative consequences of adolescent pregnancy should be a collective responsibility and concern for each and every member of the community, not only the girls, their parents or (as sometimes suggested) the President of Uganda. Findings of this study may help to direct future interventions in developing country contexts to reduce the prevalence of teenage pregnancies and assist those who become young parents.

## Data Availability

Data supporting results in this article is filed and safely locked away in the office of the Corresponding Author (Dr. Rhona Hanning) at the University of Waterloo, Ontario, Canada. The corresponding author is ready to avail the said data on reasonable request.

## Abbreviations

HIV: Human Immunodeficiency Virus
NGO: Non-Governmental Organization
ORE: Office of Research Ethics
SCT: Social Cognitive Theory
VHTs: Village Health Team workers
WHO: World Health Organization

## Footnotes

1 http://www.souluganda.org/

2 http://pefoug.org/
